# High efficiency pure blue perovskite quantum dot light-emitting diodes based on formamidinium manipulating carrier dynamics and electron state filling

**DOI:** 10.1038/s41377-022-00992-5

**Published:** 2022-12-14

**Authors:** Long Gao, Yilin Zhang, Lijie Gou, Qian Wang, Meng Wang, Weitao Zheng, Yinghui Wang, Hin-Lap Yip, Jiaqi Zhang

**Affiliations:** 1 College of Materials Science and Engineering, Key Laboratory of Automobile Materials, Ministry of Education, Jilin University, Changchun, 130012 China; 2 Femtosecond Laser laboratory, Key Laboratory of Physics and Technology for Advanced Batteries, Ministry of Education, College of Physics, Jilin University, Changchun, 130012 China; 3 Department of Materials Science and Engineering, City University of Hong Kong, Kowloon, Hong Kong China; 4 School of Energy and Environment, City University of Hong Kong, Kowloon, Hong Kong China; 5 Hong Kong Institute for Clean Energy, City University of Hong Kong, Kowloon, Hong Kong China

**Keywords:** Inorganic LEDs, Optoelectronic devices and components, Photonic devices, Quantum dots, Nanoparticles

## Abstract

Achieving high efficiency and stable pure blue colloidal perovskite quantum dot (QD) light-emitting diodes (LEDs) is still an enormous challenge because blue emitters typically exhibit high defect density, low photoluminescence quantum yield (PLQY) and easy phase dissociation. Herein, an organic cation composition modification strategy is used to synthesize high-performance pure blue perovskite quantum dots at room temperature. The synthesized FA-CsPb(Cl_0.5_Br_0.5_)_3_ QDs show a bright photoluminescence with a high PLQY (65%), which is 6 times higher than the undoped samples. In addition, the photophysical properties of the FA cation doping was deeply illustrated through carrier dynamics and first principal calculation, which show lower defects, longer lifetime, and more reasonable band gap structure than undoped emitters. Consequently, pure blue FA-CsPb(Cl_0.5_Br_0.5_)_3_ QDs light-emitting devices were fabricated and presented a maximum luminance of 1452 cd m^−2^, and an external quantum efficiency of 5.01 % with an emission at 474 nm. The excellent photoelectric properties mainly originate from the enhanced blue QDs emitter and effective charge injection and exciton radiation. Our finding underscores this easy and feasible room temperature doping approach as an alternative strategy to blue perovskite QD LED development.

## Abstract (web summary)

To gain an insight into the lattice and band structure of blue perovskite NC emitters, doping organic cations (FA^+^) reduces nonradiative recombination at room temperature. The FA^+^ significantly decreases exciton quenching and phonon energy scattering via manipulating hot carriers and electron filling in band structure.

## Graphical abstract

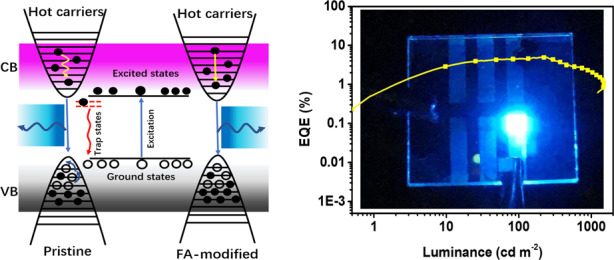

## Introduction

Perovskite halides, as a type of emerging semiconducting materials, exhibit outstanding optoelectronic properties, such as easily tunable optical bandgaps, high charge carrier mobility and long carrier diffusion length^1–5^. Benefiting from these characteristics, perovskite-based light-emitting diodes (PeLEDs) are considered as an alternative medium for high-efficiency solid-state lighting and panel display. However, the PLQYs of blue perovskite emitters, especially pure blue emission, are far behind green and red counterparts which EQEs of corresponding LEDs have both overtook 20%^6,7^. To achieve high-efficiency and high-luminance blue LEDs, devices with 3-dimensional (3D), 2D, and quasi-2D perovskites films of mixed-Cl/Br halides have been developed^8–10^. These films improve the stability of excitons and enhance the energy transfer by designing multiple-quantum-well and multi-cation-doped structure. The best-EQE device is 11.7% with an emission peak at 488 nm^11^ and 13.8 % at 496 nm^12^ so far. However, instead of thin films, perovskite QDs as emitters also show great potential in blue LEDs because of their high photoluminescence quantum yield (PLQY), strong quantum confinement effect, and high monochromaticity. Consequently, the development of blue QD emitters is still a key approach to enhance the performance of blue PeLEDs.

In 2014, the first QD-based PeLED was reported and the blue-emitting devices with Br and Cl mixed QDs were achieved with an EQE of 0.07%^13^. Then, various approaches have been employed to modify blue perovskite QDs. Ion doping has been proven a valid approach through altering the energy structure of perovskite QDs. In general, bivalent Mn^2+^, Sn^2+^, Cd^2+^, Zn^2+^ and Cu^2+^, trivalent lanthanide metal ions were often employed as B site dopants in blue perovskite QDs^14–17^. For example, the blue-emitting LED with Ni^2+^ doped CsPbX_3_ emitting at 470 nm exhibited an EQE of 2.4%^18^. In addition, a multiple-cation doping strategy, i.e., simultaneous doping of A and B sites by inorganic cations into CsPb(Br_x_Cl_3-x_), achieved high PLQY and an EQE of 2.14% for blue QD LEDs^19^. Apart from those, acid-etching small-sized QDs with low vacancy defect density and a maximum EQE value of 4.7% was realized through quantum-confined all-bromide perovskite QDs^20^.

Instead of inorganic cation doping, organic cation doping is another effective strategy to manifest blue QD emitters. Organic doping could improve the thermal, moisture, and chemical stability of QDs^21–23^. Compared with all-inorganic Cs-based perovskite QDs, partial organic cation doping may form a more stable crystal structure. For example, FA cations, a doping method for perovskite solar cells and LEDs, could tune the perovskite tolerance factor close to 1, which improves the structure stability and suppresses the ion migration. However, excellent blue QDs with FA cation doping still lack of in-depth study especially in the room temperature synthesis which currently is the most up-and-coming route for catering large-scale synthesis and commercial application of perovskite QDs.

Herein, we comprehensively study the mechanism of FA cation doped blue QDs and achieve high-efficiency pure blue QD LEDs. Formamidine acetate (FAAc) was added as a precursor for the emitters. It can strongly improve the quality of QDs to reduce defect density and the nonradiative recombination. Furtherly, FA cations affect band-edge structure and enhance the interaction of organic cations and Pb-Br octahedron frameworks. The PLQY of pure blue perovskite QDs is improved from 10% (undoped) to 65% (FA doping). The substitution manipulates the crystal growth process, grain size, carrier injection barrier, and reduces defects in perovskite QDs. Finally, we realize blue perovskite QD LEDs, which has strong EL emission peak at 474 nm corresponding color coordinates of (0.113, 0.101). And, the optimized LEDs obtained a maximum value of brightness and EQE of 1452 cd m^−2^ and 5.01%, respectively. The LEDs exhibit a T_50_ lifetime of 1056 s with an initial brightness of 100 cd m^–2^. FA cation doping is clarified to increase hot carrier relaxation and decrease nonradiative recombination by transient absorption spectroscopy. Density functional theory (DFT) calculations also elucidate that FA cations influence the state density of electrons in valence band (VB) and also the band structure, which eventually improves carrier injection.

## Results

### Structure characterization

**Fig. 1:**
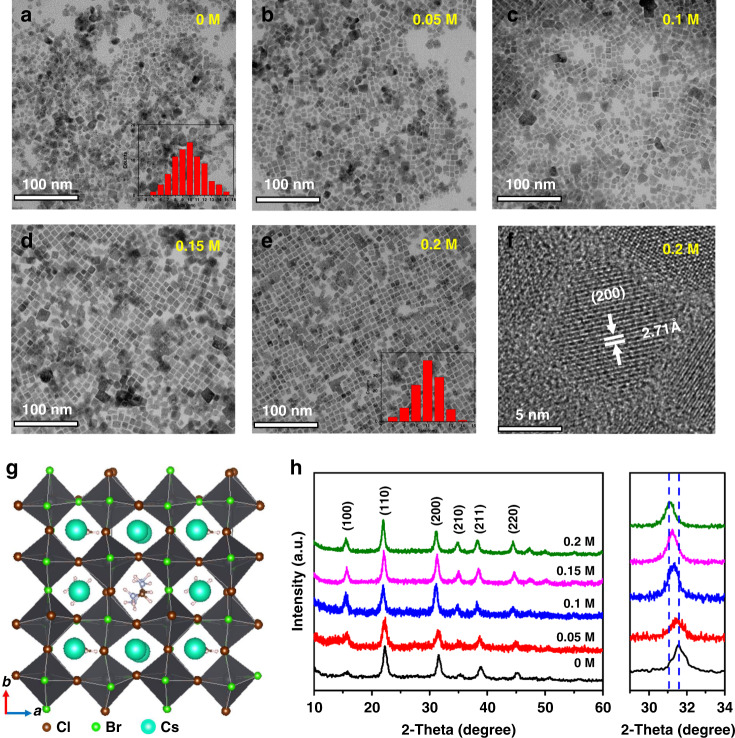
The structure and morphology of undoped and FA-doped CsPb(Cl_0.5_Br_0.5_)_3_ QDs. TEM images of **a** undoped QDs, **b** 0.05 M FA cations adding, **c** 0.1 M FA cations adding, **d** 0.15 M FA cations adding, and **e** 0.2 M FA cations adding. **f** high-resolution TEM images of 0.2 M FA cations adding. **g** the crystal structure graph. **h** XRD patterns of undoped and FA cation doped CsPb(Cl_0.5_Br_0.5_)_3_ QDs. The inserts of (**a**) and (**e**) are grain size statistical distributions

Here, the microstructure of synthetized blue perovskite QDs via room temperature ligand assisted reprecipitation method (the details are shown in Experimental Section) is shown in Fig. 1. The transmission electron microscopy (TEM) images exhibit cubic QDs of ∼11 nm for all undoped and FA-doped CsPb(Cl_0.5_Br_0.5_)_3_ QDs (Fig. 1a–e). The insets of narrower grain size distribution statistics further demonstrate the better uniformity of the cubic phase as more FA cations are added. This is mainly attributed to that the FA cations could adjust crystal framework. Clear lattice fringes were observed (Figs. 1f and S1), and the interplanar spacing of the (200) plane expands apparently from 2.60 to 2.71 Å with the adding FA cation increasing from 0 to 0.2 M, which indicates that FA cations were doped into the lattice. The schematic crystalline structure of CsPb(Cl_0.5_Br_0.5_)_3_ QDs is illustrated in Fig. 1g, where FA and Cs cations occupy the same spacing sites.

X-ray diffraction (XRD) and TEM measurements were conducted to explore the effect of FA cations on the structural properties of QDs. All samples show obvious diffraction peaks around 2θ = 15.7°, 22.3°, 31.6°, 35.2°, 38.8°, and 45.2°, corresponding to the (100), (110), (200), (210), (211), and (220) crystal planes of the cubic CsPb(Br/Cl)_3_ phase, respectively (Fig. 1h). No extra diffraction peak can be observed in the FA cation doped samples, suggesting that FA^+^ was incorporated into perovskite lattices. When the added FA cations reached 100%, the (100) diffraction peak decreases to 14.8° (Fig. S2). Also, the shift of diffraction peaks toward a lower angle suggests that the FA cations can cause lattice expansion, which is mainly due to the substitution of the smaller Cs^+^ (1.81 Å) by larger FA^+^ (2.79 Å)^24^. (200) plane was extracted as an example, in which a 0.36° shift toward a lower angle was observed with increasing FA^+^. In addition, Cs^+^ ions cause harmful shrinkage deformation of four coordination octahedrons ([PbX_6_]^4−^), which can be corrected by the doping of larger FA cations. However, excessive FA^+^ ions can cause angle increase of two adjacent coordinating octahedra (> 180°). Here, the mechanism of defect healing by FA doping could be ascribed to lattice modulation of the distortion of [PbX_6_]^4−^. Figure 1h also displays that crystal growth tendency is distinctly affected by FA cation adding. FA^+^ doped QDs realize the manipulation of crystal orientation along (100) crystal plane, which benefits to the light emission^25^.

### Photoluminescence studies and compositional analysis

**Fig. 2:**
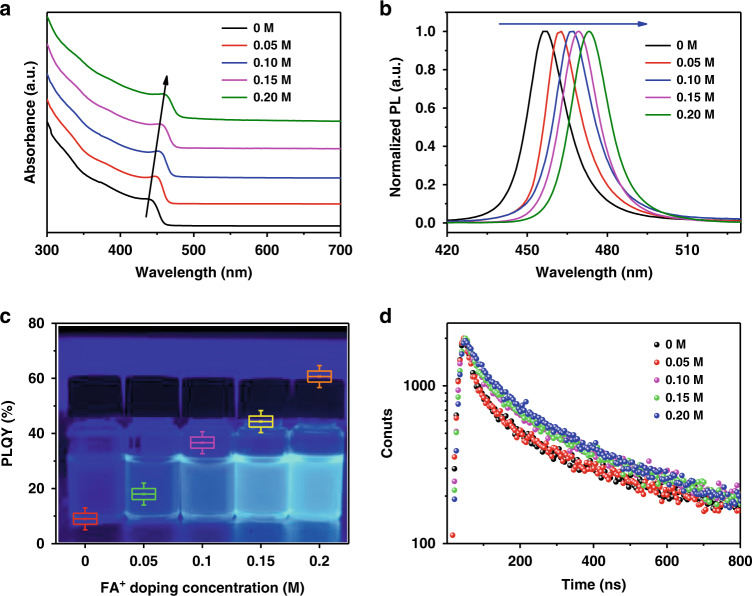
Photophysical properties of all blue QDs. **a** absorption spectra, **b** photoluminance spectra, **c** PLQY, **d** TRPL decay curves of FA^+^ doped CsPb(Cl_0.5_Br_0.5_)_3_ QDs with FA^+^ feeding ratio of 0, 0.05, 0.1, 0.15, and 0.2 M, respectively. The background inset of (**c**) shows the picture of the QDs under a UV lamp illumination with 365 nm

Figure 2 shows the optical properties of the pristine and FA^+^-doped CsPb(Cl_0.5_Br_0.5_)_3_ QDs. Compared with the pristine QDs, the absorption spectra of FA^+^-doped samples (Fig. 2a) exhibit an apparent low energy shift of the excitonic peak from 440 nm to 458 nm, and the shift finally reaches 478 nm for FA^+^-only emitters (Fig. S3a), indicating the decrease of QD optical bandgap. For investigating the origin of the bandgap change, firstly we consider the quantum size confinement of QDs, and the influence of sizes on bandgaps can be expressed by the equation^26,27^:

1 $$\Delta E = \frac{{\hbar ^2\pi ^2}}{{2m_rR^2}} - \frac{{1.786e}}{{4\pi \varepsilon _0\varepsilon R}}$$

in which *m*_r_, *R* and *ε* represent the effective mass of the excitons, the particle radius, and the relative dielectric constant of materials, respectively^28,29^. The calculation results show that the estimated maximum moving of the bandgap are around 16 meV with the particle size changing from 10 ± 0.3 nm in the pristine CsPb(Cl_0.5_Br_0.5_)_3_ QDs to 12 ± 0.4 nm in the 0.2 M FA^+^ doped CsPb(Cl_0.5_Br_0.5_)_3_ QDs, which value is much lower than the experimental change of 110 meV. Therefore, we can deduce that the doped FA cations also contribute to the change of band structure. The PL characteristics for the pristine and FA^+^ doped CsPb(Cl_0.5_Br_0.5_)_3_ QDs were further explored, as shown in Fig. 2b and Fig. S3. With the ratio of FA^+^/Cs^+^ increase, the PL peak position show red-shift (from 456 to 473 nm) and finally realize 498 nm for FAPb(Cl_0.5_Br_0.5_)_3_ (Fig. S3b).

More importantly, the PL intensity of the 0.2 M doped CsPb(Cl_0.5_Br_0.5_)_3_ QDs is obviously enhanced compared with that of pristine QDs. The absolute PLQY is illustrated in Fig. 2c and Fig. S3c, in which the PLQY of FA^+^ doped CsPb(Cl_0.5_Br_0.5_)_3_ QDs gradually increases and approaches 65% that is 6 times more than the undoped QDs. The PLQY values are listed in Table S1. The increase of PLQY primarily comes from the decreased defects in crystal structure by FA doping. To further explore the dynamic origin of the PLQY changing by FA doping, the time-resolved PL (TRPL) spectra for all samples were measured (Fig. 2d and Table S1), and the decay curves were fitted by the biexponential function. The fluorescence lifetimes are about 137.8, 154.6, 183.4, 201.0, and 214.4 ns with FA^+^ feeding ratio of 0, 0.05, 0.1, 0.15, and 0.2 M, respectively. The prolonged average lifetime indicates that nonradiative decay channels and defects are suppressed and reduced in doped samples, which improves the radiative recombination of electrons and holes and thus increases the PLQY. As a result, FA doping enhances the exciton binding energy, making excitonic emission dominates in perovskite QDs, which are shown in steady-state and time-resolved photoluminescence spectra. When the FAAC is gradually increased beyond 0.2 M (Fig. S3), the PLQY presents a peak value and then decreases, which is attributed to the excessive FA causing new defects. Simultaneously, the excessive acid in precursor solution results in the agglomeration of QDs^30^.

**Fig. 3:**
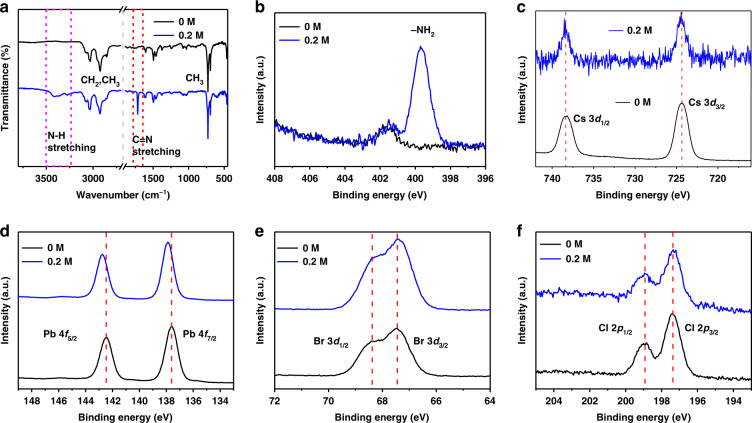
Chemical analysis of QDs. **a** FTIR spectra of pristine and FA cation doped QDs. XPS spectra of pristine and FA cation doped, N 1*s* (**b**), Cs 3*d* (**c**), Pb 4*f* (**d**), Br 3*d* (**e**), and Cl 2*p* (**f**)

To elucidate the FA cation doping, Fourier transform infrared spectroscopy (FTIR) was conducted in the pristine and treated QD samples. In Fig. 3a, both samples exhibit CH_2_ and CH_3_ symmetric and asymmetric stretching vibrations between 2840 and 2950 cm^−1^, and CH_2_ bending vibration at 1466 cm^−1^, which are the representative absorption peaks for hydrocarbon groups^2,31^. For FA-doped perovskites, a strong peak at 1716 cm^−1^ (red area) emerges, which represents the C=N stretching vibration of FA cations^32^. Subsequently, the FTIR curve for the FA-doped perovskites exhibits a broad stretching mode around 3300~3500 cm^−1^ and (pink area), which comes from the N-H stretching vibration. These vibrational peaks are obviously enhanced with the increase of FA cations (see Fig. S4 in the Supporting Information). Above data confirm that FA cations indeed doped into QDs.

We also studied the surface composition of QDs via XPS. The survey spectra of QDs confirm the existence of N, Cs, Pb, Br, and Cl elements (Fig. 3b–f and Fig. S5). Figure 3b and Fig. S5a are the high-resolution spectra of N 1*s*. The peak at 399.8 eV relates to amine groups and it originates from FA cation. The samples exhibits a weak peak at 401.8 eV for N 1*s*, which is attributed to few DDA^+^ ions from di-dodecyl dimethylammonium bromide adsorbed onto the QD surface. From Fig. 3c and Fig. S5b, the intensities of Cs 3*d* peaks are significantly weakened after FA cation doping. The results further demonstrate that FA cations partially substitute Cs cations into perovskite QDs. Furthermore, the spectra of Pb 4*f* (Fig. 3d) illustrate that the binding energies of Pb 4*f*_*5/2*_ and Pb 4*f*_*7/2*_ of the FA-doped sample are higher than those of untreated QDs. The Pb 4*f* peak position moves toward higher binding energy by 0.3 eV, which is attributed to a stronger binding between Pb and halide due to decreased octahedral volume. This also benefits to the stability of crystal structure. For the Cs 6*p*, Cl 2*p*, Br 3*d* core levels, no noticeable change was observed between two samples in high-resolution spectra.

**Fig. 4:**
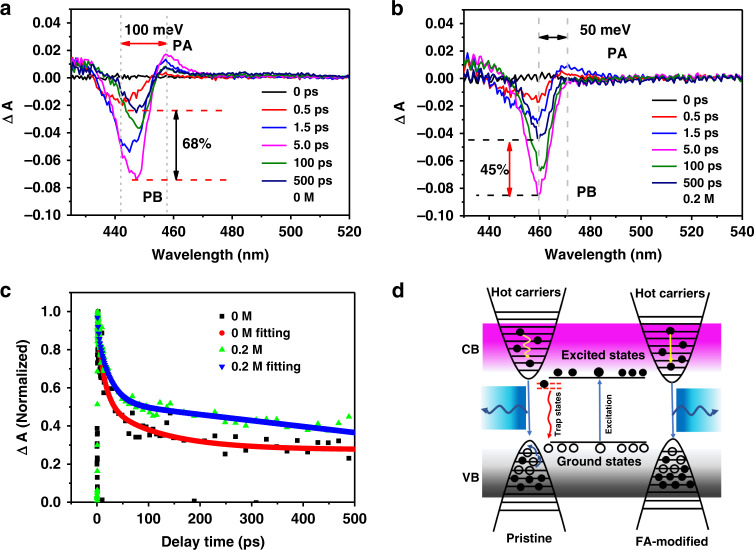
The carrier recombination dynamic of QDs. Femtosecond transient absorption spectroscopy under 400 nm pump pulse of **a** CsPb(Cl_0.5_Br_0.5_)_3_ QDs and **b** 0.2 M FA-doped CsPb(Cl_0.5_Br_0.5_)_3_ QDs; **c** the comparison of bleaching recovery kinetics of two samples (λ_ex_ = 400 nm) monitored at their bleaching maximum value; **d** the mechanism of carrier relaxation for excitation above the bandgap

In addition, the femtosecond transient absorption spectroscopy (TAS) was deducted to study the carrier dynamics and the nonradiative recombination process. The transient absorption spectra of samples were characterized under 400 nm excitation (Fig. 4 and Fig. S6). The negative signals represent photoinduced bleaching (PB) originating from the ground-state absorption, which approximate to the excitonic peaks in the absorption spectra. This is associated to the state filling of band-edge excitons (electrons and holes). The positive photoinduced absorption (PA) profiles could be attributed to the hot charge carrier absorption^33^. Comparing to Fig. 4a, Fig. 4b shows slower recoveries and stable PB peak. And for PA of both samples in Fig. 4a, b, TAS shows no contribution of PA in 0.2 M sample, indicating that doped sample process a fast hot charges carriers relaxation^34,35^. In addition, the difference between PB and PA shifts from 100 meV to 50 meV (Figs. 4a, b, and S6a–c) is attributed to the renormalization of the bandgap. Next, the bleaching recovery kinetics of the two samples are depicted in Fig. 4c. It is obvious that the short and ultrafast part is attributed to various trap-assisted nonradiative decay (carrier-phonon scattering, excitons quenching, and Auger recombination) and the improved long part is attributed to excitonic recombination process^36^. To clearly describe the kinetics process in FA cation doped CsPb(Cl_0.5_Br_0.5_)_3_ QDs, the carrier recombination mechanism is shown in Fig. 4d. The electrons in the ground states are excited by photons and then transit to the high-energy excited states. The high-energy carriers rapid cool down and reduce the scattering in electron-electron and electron-phonon. From the above investigation, excellent blue-emitting performance is attributed to the following mechanisms: (i) the fast hot charge carriers relaxation and high radiation recombination decrease energy losing; (ii) defect density was decreased to suppress non-radiation decay channel.

### Band structure

**Fig. 5:**
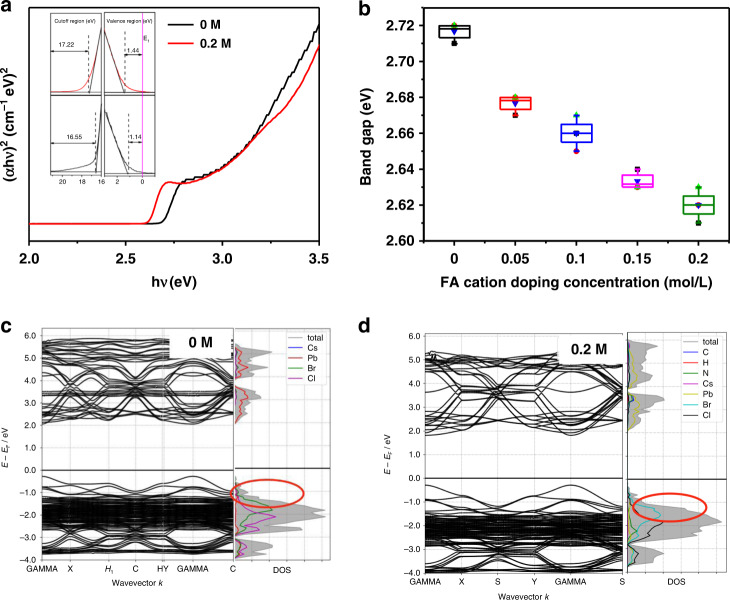
Electron state filling of QDs. **a** Tauc plots of pristine and 0.2 M FA cation doped QD films. The inset is UPS spectra of both films. **b** Optical band gap changing with FA cation increase. **c** Left: calculated band structures of pristine CsPb(Cl_0.5_Br_0.5_)_3_, and right: corresponding density of states (DOS). **d** Left: calculated band structures of FA cation doped CsPb(Cl_0.5_Br_0.5_)_3_, and right: corresponding DOS

Furthermore, we explore the influence of FA cation doping on the band structure. Firstly, we estimated the optical band gap (*E*_g_) by Tauc plots extracted from the absorption spectra, as shown in Fig. 5a and Fig. S7. The *E*_g_ value for CsPb(Cl_0.5_Br_0.5_)_3_ is 2.72 eV, which well matches with those reported elsewhere^37^. And the band gap is 2.60 eV for FA cation doped CsPb(Cl_0.5_Br_0.5_)_3_, which is > 0.1 eV larger than the undoped QDs. Furthermore, ultraviolet photoelectron spectroscopy (UPS) was conducted to explore the valence band (VB) edge positions of samples. FA cations could lead to the change of the VB edge of the QDs, as shown in Fig. 5a. The UPS data for all samples are illustrated in Fig. S8. The VB maximum energy (*E*_VB_), according to the vacuum level, was calculated to be −5.81, −5.74, −5.67, −5.62, and −5.42 eV for 0, 0.05, 0.1, 0.15, and 0.2 M FA cation doped sample, respectively. The conducting band (CB) minimum energies (*E*_CB_) can subsequently be calculated from the *E*_g_ and *E*_VB_ values. Thus, we derived the CB values for 0 and 0.2 M FA^+^ doped samples QDs as −2.81 and −3.09 eV, respectively. In addition, Fig. 5c, d show the electronic band structures and the density of states (DOS) of pristine and treated perovskites QDs, which were calculated by the first-principles. The results show that the bandgaps of CsPb(Cl_0.5_Br_0.5_)_3_ and FA-doped CsPb(Cl_0.5_Br_0.5_)_3_ are very close to each other because both CB and VB are mainly dominated by Pb and halegon ions. In the state density, VB is mainly composed of Br 3*d*, Cl 2*d* and Pb 6 *s* electrons states, while CB mainly contains Pb 6*p* electron state. The contribution of Cs and FA cations to CB and VB is negligible. However, the DOS of the doped blue QDs (Figs. 5c, d right) shows that FA cations mainly have an indirect influence on the energy band structure through manipulating halogen bonding orbits with Pb. In addition, the projected density of states (PDOS) on the C, N, H, Pb, Cs, Cl and Br atoms of both samples are computed in Fig. S9. The PDOS illustrates that the individual electronic states of FA cation mainly fill on the deep-level VB. The FA cations widen DOS band, which will cause the delocalization of carrier to decrease energy losing approach in the carrier relaxation process. The result will give a more thorough understanding of the influence of FA cations on the band structure and related carrier injection process in our devices.

### Device performance

**Fig. 6:**
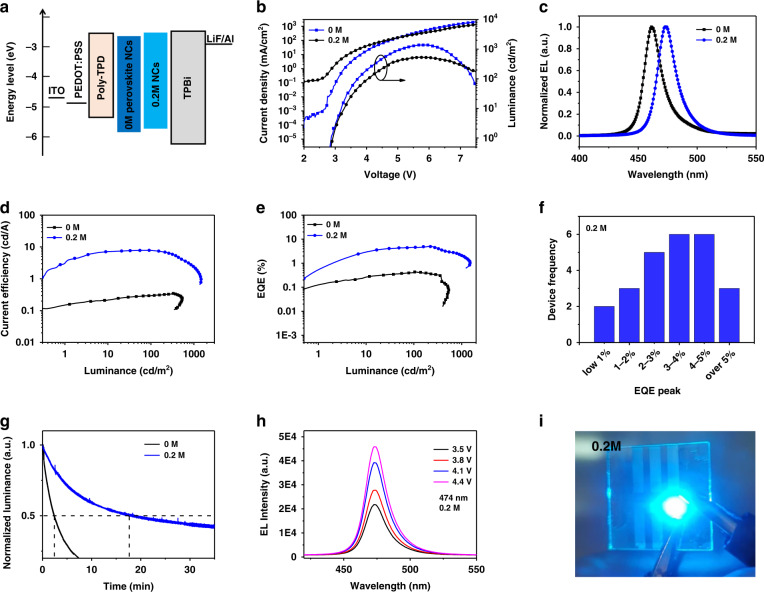
Blue LED performances based on perovskite QDs. **a** Schematic band alignment of the LED devices with 0 and 0.2 M FA cation doped QDs. **b** Curves of the current density-voltage-luminance. **c** Normalized electroluminescence spectra of the LED devices with CsPb(Cl_0.5_Br_0.5_)_3_ (black curve) and 0.2 M doped CsPb(Cl_0.5_Br_0.5_)_3_ QDs (blue curve). **d** Curves of the brightness-current efficiency. **e** Curves of the external quantum efficiency-luminance. **f** The histogram of devices frequency-EQE maximum values in 0.2 M FA cation doped CsPb(Cl_0.5_Br_0.5_)_3_ QD LEDs. **g** Emitting stability of two devices. **h** Stable EL spectra of 0.2 M FA-doped sample at different voltages. **i** The photograph of a FA-doped device operating on the voltage of 5 V

The excellent photophysical properties of FA cation doping QDs (0.2 M sample) offer exciting prospects for their exploitation in optoelectronic devices. Consequently, we fabricated pure blue LED devices with perovskite QDs acting as light-emitting layer. The schematic device energy alignment is depicted in Fig. 6a.

**Fig. 7:**
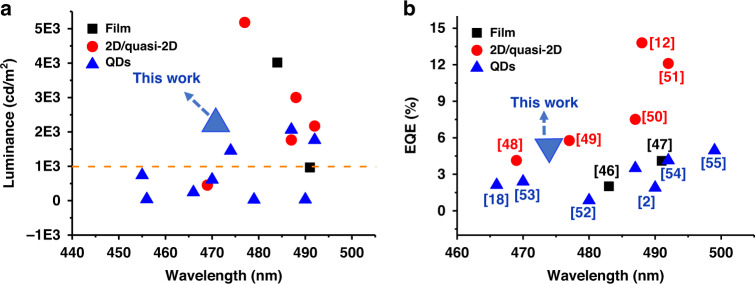
The development state of blue perovskite LEDs. The blue perovskite LEDs performance statistical graph **a** Luminance-Wavelength data and **b** EQE-Wavelength data. Ref. ^46,47^ is perovskite film LEDs, Ref. ^12,48–51^ is 2D/quasi-2D perovskite LEDs, Ref. ^2,18,52–55^ is perovskite QD LEDs

Band alignment demonstrates that FA cation doped CsPb(Cl_0.5_Br_0.5_)_3_ QD-based blue LED has a smaller barrier of hole injection than the undoped device, which changes the hole injection into the emitting layer. Figure 6b shows the voltage-dependent change of luminance and current density for two pure blue QD LEDs. The turn-on voltage (*V*_on_) (which is usually specified in literatures as the applied voltage can drive luminescence of 1 cd m^−2^) of FA-doped devices is 2.8 V, slightly smaller than that of undoped device (2.9 V). The current densities of the FA^+^ doped devices are substantially lower in the low voltage region. The peak luminance is 1452 cd m^−2^ and 522 cd m^−2^ for devices with and without FA^+^ cation doping, respectively. Figure 6c shows normalized EL spectra of two LEDs. Both EL spectra measured at 5 V and the emission wavelength are 457 and 474 nm, respectively. Coupling high luminance with low current density, the current efficiencies of FA-doped CsPb(Cl_0.5_Br_0.5_)_3_ QD LEDs show a peak value of 7.4 cd A^−1^, which is much higher than that of pure CsPb(Cl_0.5_Br_0.5_)_3_ (0.31 cd A^−1^) (Fig. 6d). Notably, the peak EQE is as high as 5.01% (Fig. 6e), which surpasses all previously reported values of pure blue CsPb(Cl_0.5_Br_0.5_)_3_ QD LEDs. The efficiencies of LEDs are dramatically improved with the introduction of FA cations (see Fig. S12 in the Supporting Information). The top performances of perovskite blue LEDs in literatures are summarized in Fig. 7 and Table S3, and here we realize the record value (5.01%) in the entire field of pure blue perovskite QD LEDs. The maximum EQE is above 10 times magnitude higher than that of the CsPb(Cl_0.5_Br_0.5_)_3_ LED. Here, FA cation doped QDs may possess better band structure to adjust charge balance. And the maximum EQE statistics are summarized in Fig. 6f, which can further illustrate device performance reliability. The high shelf stability of FA-Cs-based QD LEDs can be attributed to the excellent nanocrystals with low defect density and high PLQY. And we use half- lifetime (T_50_) to judge the device operational stability, which is set as the time needed for the device luminance to decrease to 50% of its initial value (*L*_0_). The T_50_ of the FA-based LED is about 1056 s (Fig. 6g), which is much longer than that of pristine device (150 s). In addition, EL spectrum stability of FA-based LED was measured at different voltages (Fig. 6h), and the results reveal that the QD device keeps a stable emission peak at 474 nm. All detailed parameters are shown in Table 1. And in Fig. 6i a device photo with bright pure blue emission is shown operated under 5 V.

**Table 1:** Summary of EL performance of the pristine and FA cation doped blue QD LEDs.

Device	*V*_on_^a^	Maximum value		*T*_50_^b^(s)	Peak @5 V (nm)
		L (cd m^−2^)	EQE (%)		
0	2.9	522	0.44	130	457
0.2 M	2.8	1452	5.01	1056	474

^a^ Measured voltage when luminance was 1 cd m^−2^

^b^T_50_-time when luminance decrease to 50% of initial value (100 cd m^−2^)

To further investigate the reason of the high performance of LEDs, the surface roughness of the perovskite QD films was charactered by atomic force microscopy (AFM), as shown in Fig. S13. Flat and compact surface was confirmed for the pristine and all doped samples. Good film morphology can reduce the current leakage, which is also an important factor for high performance blue perovskite LEDs. Additionally, the carrier transport properties of QD LEDs were studied by the “electron-only” and “hole-only” devices, in which current density−voltage (J−V) curves were measured (Fig. S14a, b). The device structures are as follow:

Hole-only: ITO/PEDOT:PSS/poly-TPD/QDs/MoO_3_/Al

Electron-only: ITO/PEI/ QDs/TPBi/LiF/Al

The carrier mobility of the QDs was evaluated by fitting the space charges limit current (SCLC) region with Mott−Gurney law^38^:

2 $$J_{\rm{SCLC}} = \frac{9}{8}\varepsilon _0\varepsilon _r\mu \frac{{V^2}}{{L^3}}$$

in which, ε_0_ is the vacuum dielectric constant, ε_r_ is the relative dielectric constant, μ is the mobility, V is the applied voltage and L is the thickness of the active material. The hole mobilities of undoped and 0.2 M FA cation doped QDs films are 6.24 × 10^−6^ and 1.32 × 10^−4^ cm^2^ V^−1^ s^−1^, respectively. The electron mobilities of CsPb(Cl_0.5_Br_0.5_)_3_ and FA cation doped CsPb(Cl_0.5_Br_0.5_)_3_ QDs films are 3.78 × 10^−4^ and 8.20 × 10^−5^ cm^2^ V^−1^ s^−1^, respectively. With the adding of FA cations, the electron mobility decreases while the hole mobility increases, which reveals that the mobilities of two carrier species are more balanced with QD modification and this result agrees with the aforementioned UPS data. In addition, balanced carrier mobility could decline emitting quenching.

## Discussion

We successfully realized FA cation doped pure blue CsPb(Cl_0.5_Br_0.5_)_3_ QDs at room temperature. The FA cation doping manipulate the morphology and light emission of QDs. It boosts PLQY from 10% to 65% by decreasing nonradiative recombination. The fluorescence lifetime increases 1.6 times than the undoped ones. TAS further elaborates the mechanism of excellent QD emitters originating from fast carrier relaxation and low defects to decrease energy losing channels of QDs. Simultaneously, the first-principle demonstrates that electronic state density of valence band is changed to decline the carrier injection barriers. Ultimately, a champion device was obtained with a high luminance and a peak EQE of 1452 cd m^−2^ and 5.01% at 474 nm, respectively. This work offers a good approach to develop Cl/Br mixed room temperature-synthesized pure blue-emitting perovskite QDs.

## Materials and methods

### Materials

Cs_2_CO_3_(99.9%), didodecyldimethylammonium bromide (DDAB,98%), toluene (ACS grade, Fisher), Octanoic acid (OTAc,98%), Formamidine acetate (FAAc, 99%), tetraoctylammonium bromide (TOAB,98%), Methyl acetate (98%), and aluminum (Al) were purchased from Sigma-Aldrich. PbBr_2_ (99.9%), PbCl_2_ (99.9%), Poly(3,4-ethylenedioxythiophene)-poly(styrenesulfonate) dry re-dispersible pellets (PEDOT:PSS (4083)), Poly[N,N’-bis(4-butylphenyl)-N,N’-bis(phenyl)-benzidine (Poly-TPD), 1,3,5-Tris(1-phenyl-1H-benzimidazol-2-yl)benzene(TPBi), and LiF were purchased from Xi’an Polymer Light Technology Corp.

### **Synthesis and purification FAAc doped CsPb(Cl**_**0.5**_**Br**_**0.5**_**)**_**3**_**QDs**

The CsPb(Cl_0.5_Br_0.5_)_3_ QDs were synthesized by referencing double ligand-assisted-reprecipitation methods^39^ with some modifications. First, cesium precursor was prepared by loading 0.5 mmol of Cs_2_CO_3_ and 5 mL of OTAc into a 20 mL bottle, and then 0.25 mmol, 0.5 mmol, 0.75 mmol, and 1 mmol FAAc were added and stirred for 20 min at room temperature. 1 mmol mixture of PbBr_2_ and PbCl_2_ was added to 50 ml flask. Then, 2 mmol of TOAB and 10 ml toluene were also filled into the bottle to form precursor solution. For the synthesis of pure CsPb(Cl_0.5_Br_0.5_)_3_ QDs, 1.0 mL of a Cs^+^ precursor solution was swiftly added into 9 mL of a PbX_2_ toluene solution. The solution was magnetically stirred for 10 min at room temperature in open air. Subsequently, 3 mL of DDAB (in toluene 10 mg mL^−1^) solution was added. After 1 min, a volume ratio of 2:1 for ethyl acetate was put into the crude solution; the precipitates were collected separately after centrifugation and dispersed in toluene. The additional ethyl acetate was put into the dispersion solution, and the precipitates were collected and re-dispersed in 2 ml toluene. As for FA-doped QDs, the different mass of FAAc was added into Cs^+^ precursor to form mixture A-site cations, and the other processes were the same.

### **LED fabrication**

Pre-patterned indium tin oxide (ITO) glasses with an 8 Ω/square sheet resistance were used as the substrates for the blue QD LEDs. Deionized water, acetone, and isopropanol were used to sequentially clean the ITO substrates. Then the substrates were exposed to UV–ozone ambiance for 5 min at 50 W before sequential coating. The lighting active area of QDs LEDs was 2 × 2 mm^2^. The detailed device structure was ITO/ PEDO:PSS/ Poly-TPD/QDs/TPBi (50 nm)/LiF (1 nm)/Al (100 nm), which was reported elsewhere^40^. PEDOT:PSS and Poly-TPD were as hole transport layers. PEDOT:PSS was spin-coated and then annealed in air at 120 °C for 20 min to form a 30 nm layer. Next, Poly-TPD film was spin-coated and baked at 120 °C for 15 min in a glove box to form a 20 nm layer. Then CsPb(Cl_0.5_Br_0.5_)_3_ and FA cation doped QD solutions were spin coated on the smooth poly-TPD film at 2000 rpm for 60 s, and baked at 50 °C for 10 min to form a 40 nm layer. The remaining layers (TPBi, LiF, Al) were deposited in a thermal evaporator with a pressure of 5 × 10^−4^ Pa, which deposition rates were 0.2, 0.01, 1 Å s^−1^, respectively. Film thickness and evaporation rate were controlled by a quartz-crystal sensor.

### **First-principles calculations**

The electron structures of pristine and FA cation doped CsPb(Cl_0.5_Br_0.5_)_3_ were conducted using the Vienna Ab initio Simulation Package code^41–43^. The projector augmented wave (PAW) approach and the generalized gradient approximation of Perdew, Burke, and Ernzerhof (PBE) describe the ion-electron interactions and exchange-correlation function^44,45^. For all calculations, the energy cut-off of 520 eV for the plane-wave basis was used with k-points meshes of spacing 2π × 0.03 Å. All structures were fully optimized until the total energy and residual forces of each atom converged to 10 eV and were smaller than 10 eV Å^−1^, respectively.

### **Characterization techniques**

Transmission electron microscope (TEM, FEI Tecnai F20) were used to study lattice sizes of the perovskite QDs samples. A Bruker D8 X-ray diffractometer which used a copper Kα radiation (λ = 1.54178 Å) characterized the film X-ray diffraction (XRD). A Cary Eclipse spectrofluorometer show PL spectra of QDs emitter. A PerkinElmer Lambda 3600 UV–vis–NIR spectrometer test ab-sorption curves. Time-resolved PL (TRPL) data were recorded by using the Edinburgh FLS980 spectrofluorometer with a 405 nm laser. PLQY was as well as tested by the same fluorescence spectrometer with an integrating sphere. A Nicolet 6700 FT-IR spectrometer was used to perform Fourier transform infrared spectra (FTIR). The X-ray photoelectron spectroscopy (XPS) was col-lected through ESCALAB 250 X-ray photoelectron spectrometer. A Keithley 2612 source meter connecting with a Newport 818-UV Si photodiode tested current-voltage-luminance characteristics. A NOVA spectrometer recorded EL spectra.

## Supplementary information

Calculation perovskite tolerance factor and decay lifetime, characteristic parameters, HRTEM, XRD, UV–vis absorbance, PL, PLQY, UPS, Tauc plots, PL stability, SEM cross-section imgine, AFM, Partial state density, J–V curves of single-carrier devices, and Lambertian profile. This material is available free of charge at http://xxx.
