# Supplementary material for: High efficiency pure blue perovskite quantum dot light-emitting diodes based on formamidinium manipulating carrier dynamics and electron state filling
Source: Light Sci Appl. 2022 Dec 14;11:346. doi: 10.1038/s41377-022-00992-5 (PMC9747997; doi:10.1038/s41377-022-00992-5)
Supplement: Supplementary file 1 — Calculation perovskite tolerance factor and decay lifetime, characteristic parameters, HRTEM, XRD, UV–vis absorbance, PL, PLQY, UPS, Tauc plots, PL stability, SEM cross-section imgine, AFM, Partial state density, J–V curves of single-carrier devices, and Lambertian profile. This material is available free of charge at http://xxx. [file 41377_2022_992_MOESM1_ESM.pdf]

Supplementary Information

High efficiency pure blue perovskite quantum dots light emitting diodes based on formamidinium manipulating carrier dynamics and electron state filling

Long Gao,[1] Yilin Zhang,[1] Lijie Gou,[1] Qian Wang,[1] Meng Wang, [1] Xiaoyu Zhang,[1] Weitao Zheng,[1] Yinghui Wang[2], Hin-Lap Yip[3,4,5]*, Jiaqi Zhang[1]*

[1] College of Materials Science and Engineering, Key Laboratory of Automobile Materials, Ministry of Education, Jilin University, Changchun, 130012, China
[2] Femtosecond Laser laboratory, Key Laboratory of Physics and Technology for Advanced Batteries, Ministry of Education, College of Physics, Jilin University, Changchun 130012, China.
[3] Department of Materials Science and Engineering, City University of Hong Kong, Kowloon, Hong Kong
[4] School of Energy and Environment, City University of Hong Kong, Kowloon, Hong Kong
[5] Hong Kong Institute for Clean Energy, City University of Hong Kong, Kowloon, Hong Kong

Corresponding author's email address: zhangjiaqi@jlu.edu.cn; a.yip@cityu.edu.hk

Calculation tolerance factor via ionic radius

One major structural aspect is that partial substitution at the A-site influences the Goldschmidt's tolerance factor[1],

$$t = \frac{R_X + R_A}{\sqrt{2}\,(R_X + R_B)} \qquad \text{Equation S1}$$

where RA is the radius of the A-site cations, RB is the radius of the B-site cations, and RX is the radius of the anions. The effective ionic radii: R(Cs^+) = 169 pm, R(Pb^{2+}) = 119 pm, R(FA^+) = 235 pm, R(Cl^-) = 181 pm, and R(Br^-)=195 pm.[2] For a certain composition of FA-$CsPb(Cl_{0.5}Br_{0.5})_3$ QDs, effective R_A ($R_{A'}$) and tolerance factors (t') are calculated by following formulas:

$$R'_A = (1-x)167 + 235x, (0 \le x \le 1) \qquad \text{Equation S2}$$

$$R'_X = 0.5 \times 181 + 195 \times 0.5 = 188\,pm$$

$$t' = \frac{R'_X + R'_A}{\sqrt{2}\,(R'_X + R_B)} \qquad \text{Equation S3}$$

When the FA ratio changes among 0, 0.05, 0.1, 0.15 and 0.2 M FA^+, the t' increased from 0.822 to 0.859, 0.886, 0.905 and 0.919, respectively, illustrating that incorporation of FA cation improves the structural stability, which further supported our experimental results.

Calculation of decay lifetime and radiative & non-radiative rate

The PL decay curves can be fitted with a bi-exponential function[3–5]:

$$I = A_1 \exp\left(\frac{-t}{\tau_1}\right) + A_2 \exp\left(\frac{-t}{\tau_2}\right) \qquad \text{Equation S4}$$

where I is the luminescence intensity at time t, A_1 and A_2 are weight constants, and τ_1 and τ_2 are the time constants for the exponential components. The average PL lifetime (τ_{av}) can be determined by the expression:

$$\tau_{av} = (A_1\tau_1^2 + A_2\tau_2^2)/(A_1\tau_1 + A_2\tau_2) \qquad \text{Equation S5}$$

Radiative lifetime:

$$\tau_r = \frac{\tau_{ave}}{PLQY} \qquad \text{Equation S6}$$

Nonradiative lifetime:

$$\tau_{nr} = \frac{\tau_{ave}}{1-PLQY} \qquad \text{Equation S7}$$

The radiative decay rate was got by Eq. S6 and the nonradiative decay rate was got by

Eq. S7. The average PL lifetimes can be calculated.

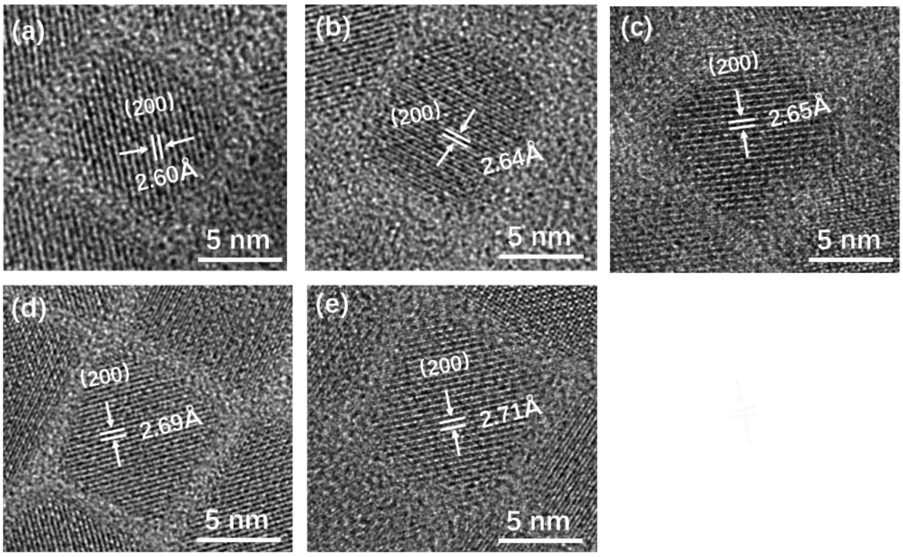

Fig. S1. High resolution TEM images of 0, 0.05, 0.1, 0.15, and 0.2 M FA cation doped samples.

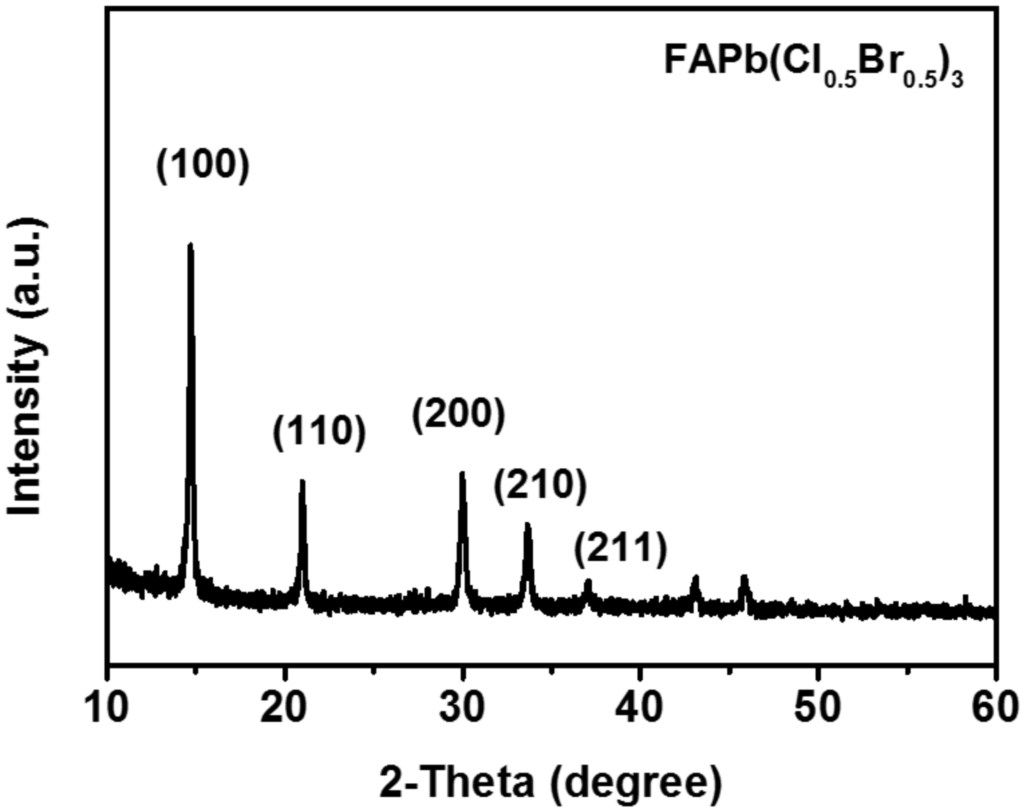

Fig. S2. XRD pattern of $FAPb(Cl_{0.5}Br_{0.5})_3$ QD film.

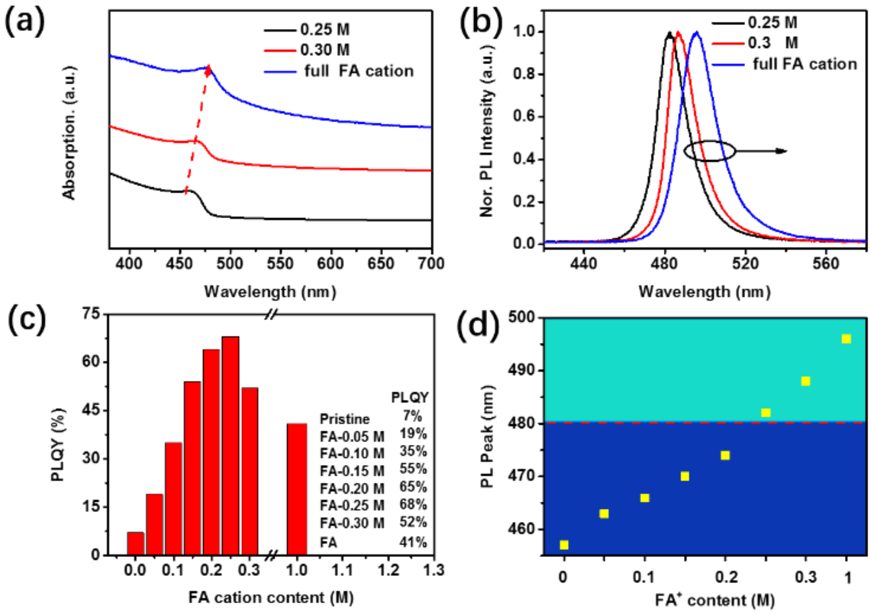

Fig. S3. (a) UV−vis absorption spectra of QDs with 0.25 M, 0.3 M, and 100% FA^+ doped samples. (b) Photoluminescence spectra of QDs with 0.25 M, 0.3 M, and 100% FA^+ doping. (c) The PLQY values with the increasing of FA cation doping amount. (d) PL peak changing with increasing of FA cation doping amount.

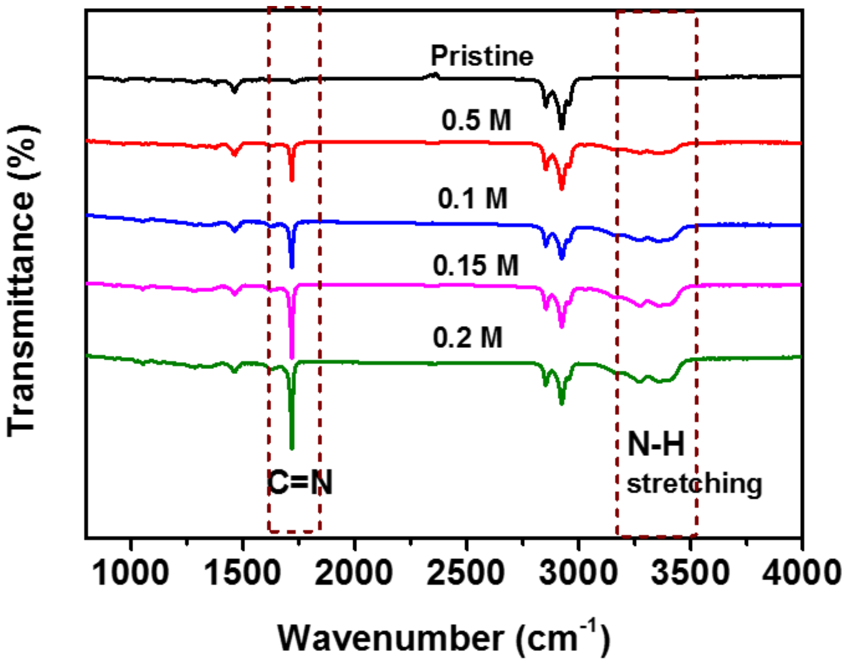

Fig. S4. (a) FTIR spectra of pristine, 0.05 M, 0.1 M, 0.15 M and 0.2 M FA cation doped perovskite QDs.

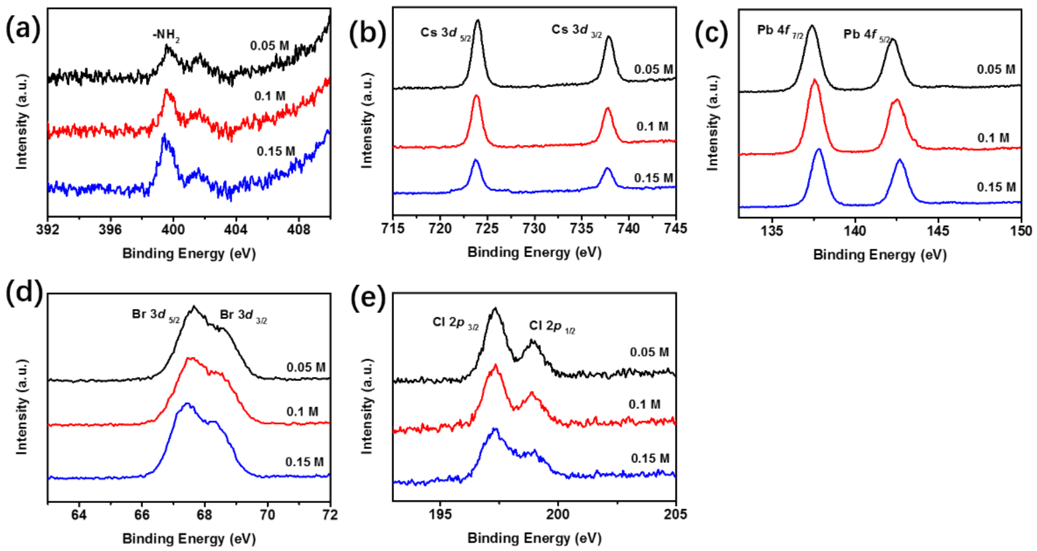

Fig. S5. XPS spectra of N 1s (a), Cs 3d (b), Pb 4f (c), Br 3d (d), and Cl 2p (c) for 0.05 M, 0.1 M, and 0.15 M FA cation doped QDs.

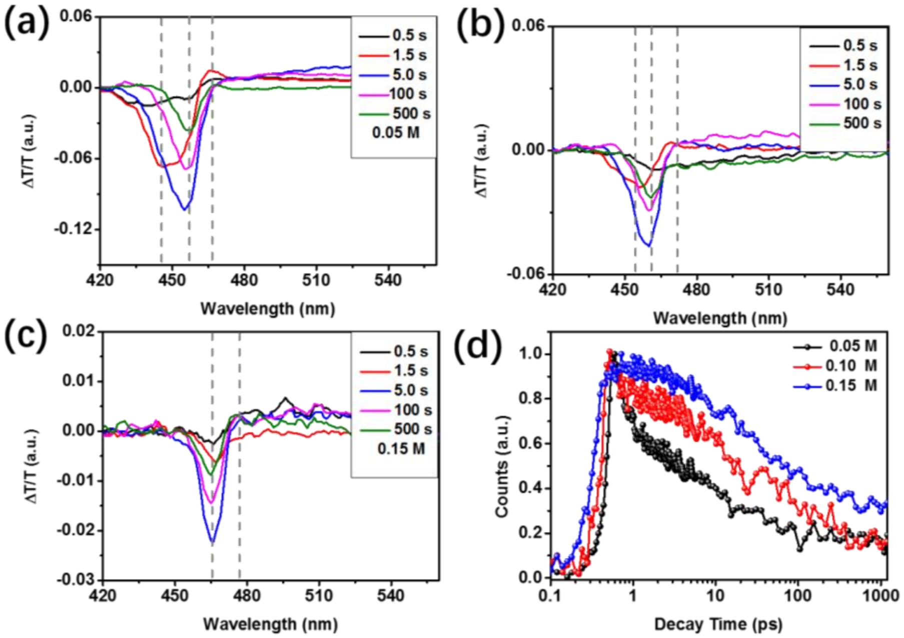

Fig. S6. Transient absorption spectroscopy of $CsPb(Cl_{0.5}Br_{0.5})_3$ QDs with FA cation doping for 0.05 M (a), 0.1 M (b), and 0.15 M (c). The kinetics (d) of the three bleaches QDs.

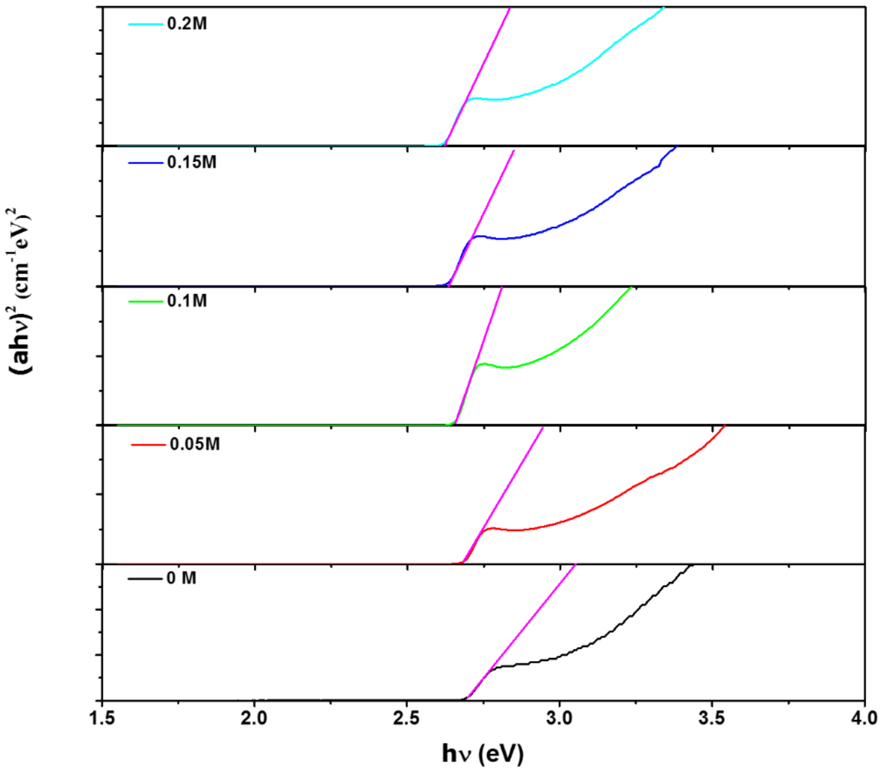

Fig. S7. Tauc plot of $CsPb(Cl_{0.5}Br_{0.5})_3$ QDs films with 0, 0.05, 0.1, 0.15, and 0.2 M FA^+ doped samples on quartz substrates.

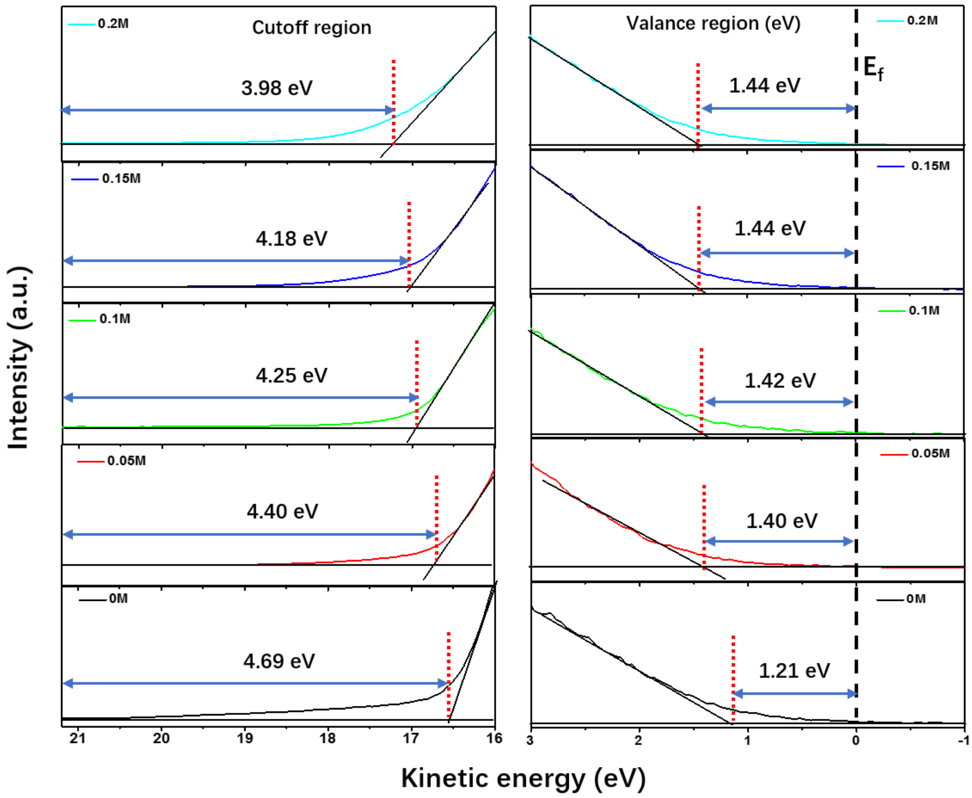

Fig. S8. UPS spectra of QDs film with 0, 0.05, 0.1, 0.15, and 0.2 M FA^+ doped samples on silicon substrates.

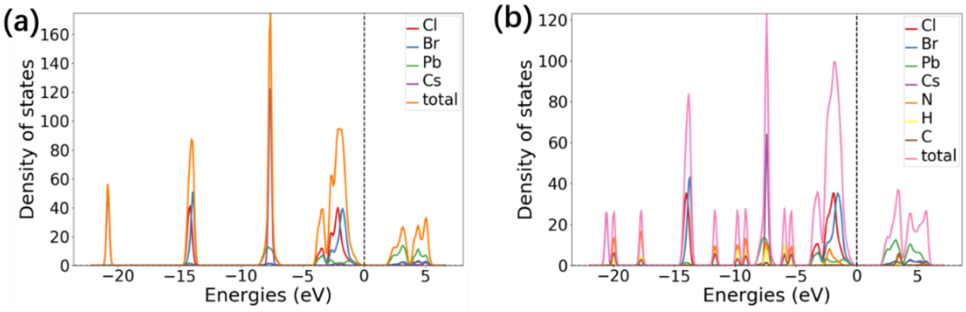

Fig. S9. The partial densities of states of the projected atomic states of C, N, H, Pb, Cs, Cl and Br orbitals for (a) $CsPb(Cl_{0.5}Br_{0.5})_3$ QDs and (b) 0.2 M FA cation doped samples.

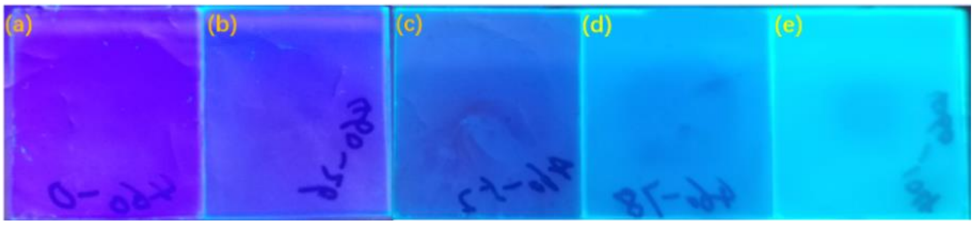

Fig. S10. Fluorescence photos of pristine, 0.05, 0.1, 0.15, and 0.2 M FA^+ doped perovskite QD films on quartz substrates.

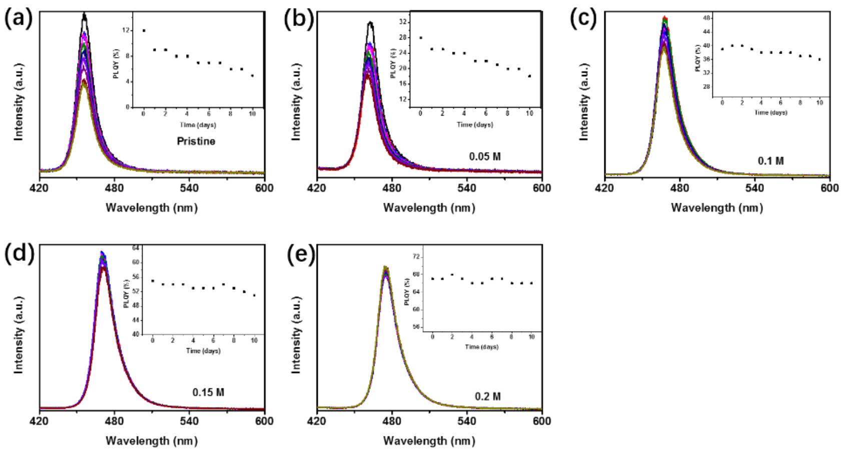

Fig. S11. PL spectra intensity changing of all samples at different time intervals and insets correspond to PLQY value.

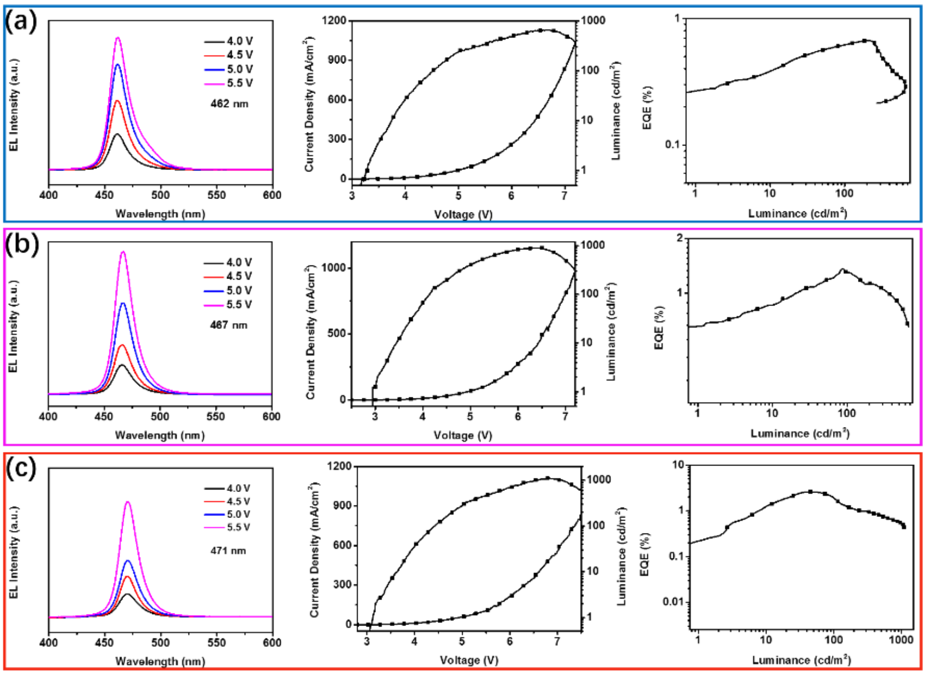

Fig. S12. EL spectra at applied voltage from 4.0 - 5.5 V, Current density (J) and brightness (L) vs driving voltage (V) and EQE vs L, (a) present 0.05 M FA^+ doped device, (b) present 0.1 M FA^+ doped device, and (c) present 0.15 M FA^+ doped device.

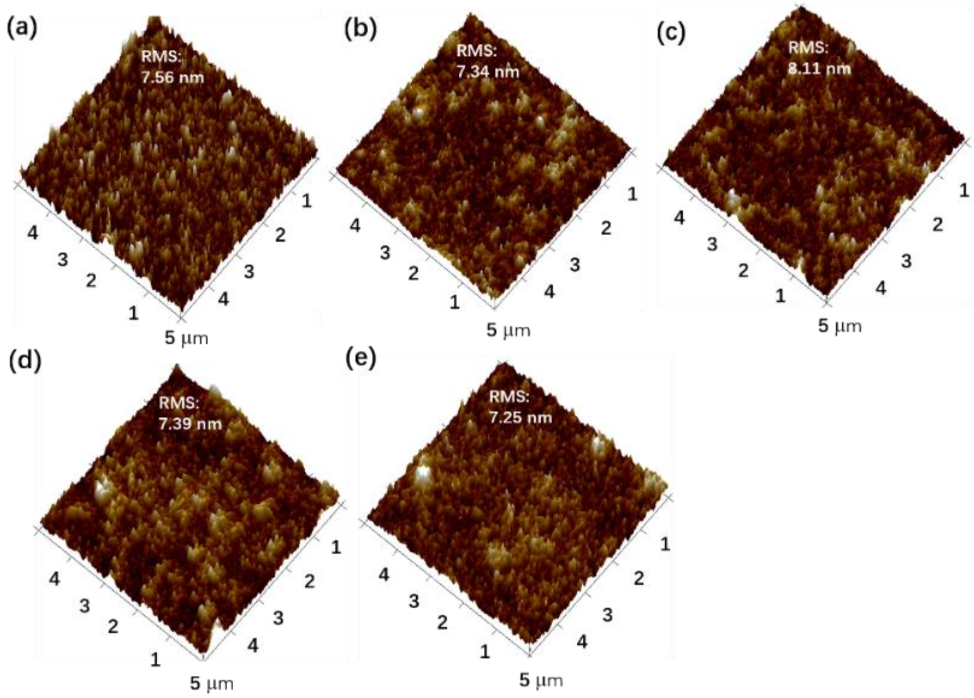

Fig. S13. AFM images for pristine (a), 0.05 M FA^+ doped (b), 0.1 M FA^+ doped (c), 0.15 M FA^+ doped (d), and 0.2 M FA^+ doped (e) $CsPb(Cl_{0.5}Br_{0.5})_3$ perovskite QD films.

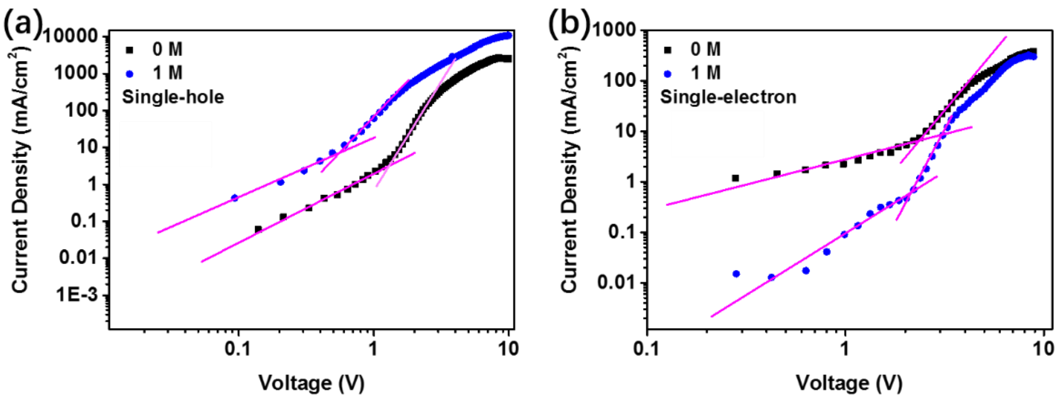

Fig. S14. J -V curves of hole-only devices (a) and electron-only devices (b).

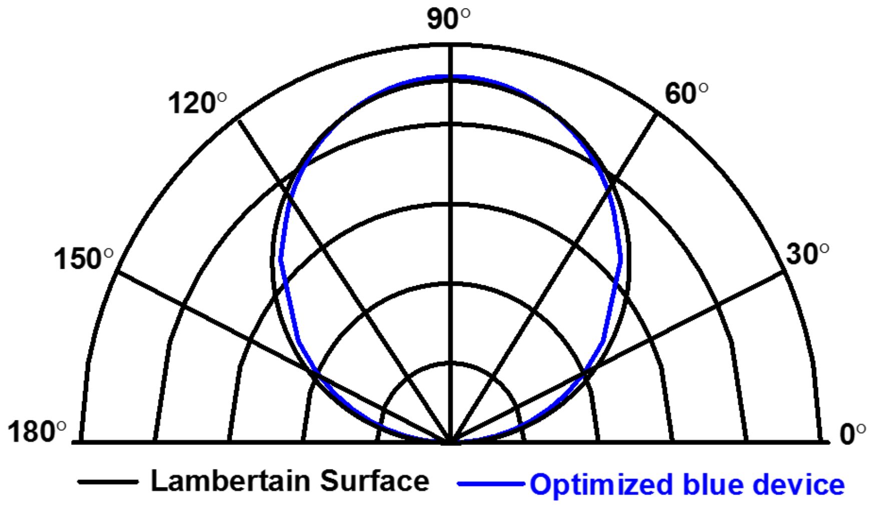

Fig. S15. Angular distribution EL radiation intensity follows the Lambertian profile.

Table S1. The optical and physical property parameters of QDs

Samples	0 FA^+	0.05 M FA^+	0.1 M FA^+	0.15 M FA^+	0.2 M FA^+
Peak (nm)	456	463	466	469	473
PLQY (%)	10	16	35	44	65
A_1	1041.06	1139.96	986.64	780.52	636.67
t_1 (ns)	19.51	22.63	28.30	29.07	35.22
A_2	777.03	737.23	888.34	1006.84	1111.27
t_2 (ns)	157.40	180.25	208.13	218.80	230.74
T_{ave} (ns)	137.76	154.62	183.44	200.99	214.37

Table S2. The atoms statistics results for XPS

QDs	N	Cs	Pb	Cl	Br
$CsPb(Cl_{0.5}Br_{0.5})_3$	0	20.33	21.42	28.10	30.15.
0.05M-FA:$CsPb(Cl_{0.5}Br_{0.5})_3$	3.91	17.63	20.28	27.93	30.26
0.1M-FA:$CsPb(Cl_{0.5}Br_{0.5})_3$	7.05	15.47	19.83	26.76	30.89
0.15M-FA:$CsPb(Cl_{0.5}Br_{0.5})_3$	9.58	12.46	18.54	27.85	31.64
0.2M-FA:$CsPb(Cl_{0.5}Br_{0.5})_3$	11.85	9.22	18.68	28.30	31.95
$FAPb(Cl_{0.5}Br_{0.5})_3$	22.19	0	19.02	27.92	30.87

Table S3. Summary of EL performance of typical blue-emissive halide perovskite LEDs

Blue perovskite devices	V_{on}	L(cd m^{-2})	EQE (%)	Peak (nm)	T_{50} (min)	Ref.
3D film	3.0	4015	2.01	484	300 min	Ref.[6]
	3.0	968.1	4.1	491	60 min	Ref.[7]
quasi-2D and 2D film	3.5	1315	3.08	445	200 s	Ref.[8]
	3.3	451	4.14	469	14 min	Ref.[9]
	4	3780	5.2	479	90 min	Ref.[10]
	3.2	5183	5.6	477	120 min	Ref.[11]
	3	513	10.11	486	81 min	Ref.[12]
	3.4	2170	11.7	492	900 s	Ref.[13]
	2.6	2191	12.1	488	---	Ref.[13]
	2.7	3000	13.8	488	13 min	Ref.[14]
QDs	5.1	742	0.07	455	---	Ref.[15]
	3.5	30	0.86	480	60 s	Ref.[16]
	3.0	34	1.9	490	---	Ref.[17]
	5.5	43.2	1.1	456	---	Ref.[18]
	4	245	2.12	466	---	Ref.[19]
	4.2	620	2.15	470	---	Ref.[20]
	3.2	612	2.4	470	---	Ref.[21]
	2.75	1762	4.14	492	105 s	Ref.[22]
	3.2/3.0	2063/7600	3.5/4.96	487/499	---	Ref.[23]
**This work**	**2.8**	**1452**	**5.01**	**474**	**17.6 min**	**---**

References

1. Chen, J. & Park, N.-G. Materials and methods for interface engineering toward stable and efficient perovskite solar cells. *ACS Energy Lett.* 2742–2786 (2020).
2. Park, B. wook & Seok, S. Il. Intrinsic instability of inorganic–organic hybrid halide perovskite materials. *Adv. Mater.* **31**, 1–17 (2019).
3. Yang, F. *et al.* Efficient and spectrally stable blue perovskite light-emitting diodes based on potassium passivated nanocrystals. *Adv. Funct. Mater.* **30**, 1–7 (2020).
4. Cho, H. *et al.* Overcoming the electroluminescence efficiency limitations of perovskite light-emitting diodes. *Science.* **350**, 1222–1225 (2015).
5. Li, J. *et al.* 50-fold EQE improvement up to 6.27% of solution-processed all-inorganic perovskite $CsPbBr_3$ QLEDs via surface ligand density control. *Adv. Mater.* **29**, (2017).
6. Yuan, F. *et al.* A cocktail of multiple cations in inorganic halide perovskite toward efficient and highly stable blue light-emitting diodes. *ACS Energy Lett.* **5**, 1062–1069 (2020).
7. Li, J. *et al.* Strontium ion B-site substitution for spectral-stable blue emitting perovskite light-emitting diodes. *Adv. Opt. Mater.* **8**, 1–9 (2020).
8. Yan, S. *et al.* Deep blue layered lead perovskite light-emitting diode. *Adv. Opt. Mater.* **9**, 1–9 (2021).
9. Shen, Y. *et al.* Interfacial potassium-guided grain growth for efficient deep-blue perovskite light-emitting diodes. *Adv. Funct. Mater.* **31**, 2006736 (2021).
10. Ma, D. *et al.* Chloride insertion-immobilization enables bright, narrowband, and stable blue-emitting perovskite diodes. *J. Am. Chem. Soc.* **142**, 5126–5134 (2020).
11. Wang, Q. *et al.* Efficient sky-blue perovskite light-emitting diodes via photoluminescence enhancement. *Nat. Commun.* **10**, (2019).
12. Ren, Z. *et al.* High performance blue perovskite light-emitting diodes enabled by efficient energy transfer between coupled quasi-2D perovskite layers. *Adv. Mater.* **33**, 1–10 (2021).

13. Pang, P. *et al.* Rearranging low-dimensional phase distribution of quasi-2D perovskites for efficient sky-blue perovskite light-emitting diodes. *ACS Nano* **14**, 11420–11430 (2020).

14. Zhu, Z. *et al.* Highly efficient sky-blue perovskite light-emitting diode via suppressing nonradiative energy loss. *Chem. Mater.* **33**, 4154–4162 (2021).

15. Song, J. *et al.* Quantum dot light-emitting diodes based on inorganic perovskite cesium lead halides ($CsPbX_3$). *Adv. Mater.* **27**, 7162–7167 (2015).

16. Shin, Y. S. *et al.* Vivid and fully saturated blue light-emitting diodes based on ligand-modified halide perovskite nanocrystals. *ACS Appl. Mater. Interfaces* **11**, 23401–23409 (2019).

17. Pan, J. *et al.* Highly efficient perovskite-quantum-dot light-emitting diodes by surface engineering. *Adv. Mater.* **28**, 8718–8725 (2016).

18. Chiba, T. *et al.* Blue perovskite nanocrystal light-emitting devices via the ligand exchange with adamantane diamine. *Adv. Opt. Mater.* **8**, 2000289 (2020).

19. Hou, S. *et al.* Efficient blue and white perovskite light-emitting diodes via manganese doping. *Joule* **2**, 2421–2433 (2018).

20. Shao, H. *et al.* High brightness blue light-emitting diodes based on $CsPb(Cl/Br)_3$ perovskite QDs with phenethylammonium chloride passivation. *Nanoscale* **12**, 11728–11734 (2020).

21. Pan, G. *et al.* Bright blue light emission of Ni^{2+} ion-doped $CsPbCl_xBr_{3-x}$ perovskite quantum dots enabling efficient light-emitting devices. *ACS Appl. Mater. Interfaces* **12**, 14195–14202 (2020).

22. Zhang, F. *et al.* Stabilizing electroluminescence color of blue perovskite LEDs via amine group doping. *Sci. Bull.* **66**, 2189–2198 (2021).

23. Shynkarenko, Y. *et al.* Direct synthesis of quaternary alkylammonium-capped perovskite nanocrystals for efficient blue and green light-emitting diodes. *ACS Energy Lett.* **4**, 2703–2711 (2019).
